# Coronary Flow Reserve in Pregnant Rats with Increased Left Ventricular Afterload

**DOI:** 10.1371/journal.pone.0102147

**Published:** 2014-07-09

**Authors:** Nils Thomas Songstad, Maria C. Serrano, Vasilis Sitras, Davis Johansen, Kirsti Ytrehus, Ganesh Acharya

**Affiliations:** 1 Women's Health and Perinatology Research Group, Institute of Clinical Medicine, Faculty of Health Sciences, UiT – The Arctic University of Norway, Tromsø, Norway; 2 Department of Pediatrics, University Hospital of Northern Norway, Tromsø, Norway; 3 Division of Pediatric Cardiology, Department of Pediatrics, University of Miami, Jackson Memorial Hospital, Miami, Florida, United States of America; 4 Department of Obstetrics and Gynecology, University Hospital of Northern Norway, Tromsø, Norway; 5 Cardiovascular Research Group, Department of Medical Biology, Faculty of Health Science, UiT – The Arctic University of Norway, Tromsø, Norway; University of Otago, New Zealand

## Abstract

**Background:**

Coronary flow reserve (CFR) is used as a measure of coronary endothelial function. We investigated the effect of increased afterload on CFR of pregnant and non-pregnant rats.

**Methods:**

Afterload increase in Wister rats (both pregnant and non-pregnant) was achieved by the infusion of angiotensin II (Ang II) for ∼10 days or by subjecting them to transverse aortic constriction (TAC) for ∼14 days. Control groups were infused with 0.9% NaCl or had sham surgery, respectively. In pregnant rats, the experiments were performed close to term gestation. Doppler velocity waveforms of the left main coronary artery were recorded using a high resolution ultrasound imaging system (Vevo 770, VisualSonics, Canada) at baseline while the animals were anesthetized with 1.5% inhaled isoflurane, and during maximal coronary dilatation obtained by the inhalation of 3.5% of isoflurane. CFR was calculated as the ratio between the peak coronary flow velocities (CFR_peak_) and the velocity-time integrals (CFR_VTI_) recorded at hyperemia and at baseline.

**Results:**

CFR could be calculated in 60 of 75 (80%) animals. There were no differences in CFR between intervention and control groups irrespective of whether afterload was increased by Ang II or TAC. In the TAC-study CFR_peak_ (1.54±0.07 vs 1.85±0.17; p = 0.03) was decreased in pregnant compared to non-pregnant shams. When sham animals from both studies were pooled together both CFR_peak_ (1.42±0.07 vs 1.86±0.16; p = 0.005) as well as CFR_VTI_ (1.45±0.07 vs 1.78±0.12; p = 0.03) were significantly lower in pregnant rats compared to non-pregnant.

**Conclusions:**

CFR can be measured non-invasively in rats using Doppler echocardiography and high concentrations of inhaled isoflurane as a coronary vasodilator. In pregnant rats, CFR is reduced close to term. CFR is not affected by increased left ventricular afterload caused by chronic Ang II infusion or TAC.

## Introduction

Coronary flow reserve (CFR), i.e. the ratio of maximum to baseline coronary blood flow [1], is used as a measure of coronary endothelial function. Human studies have shown a link between impaired coronary microvascular function and adverse cardiovascular events [2], [3]. In order to measure CFR, application of an agent with potent endothelium independent vasodilating properties is needed. Adenosine administered intravenously is usually preferred due to its rapid action and short half time [1], [4], and has been used in both human [5], [6] and animal studies [7], [8]. Inhaled isoflurane, which is used as an anaesthetic in small animal research, is a potent coronary vasodilator. Hartley et al. have shown that the increase in coronary blood flow after administering high concentrations of inhaled isoflurane can be used to estimate CFR in mice [9], [10].

Normal pregnancy is characterised by increased cardiac preload and decreased afterload [11], [12]. However, in hypertensive disorders of pregnancy, and in some women with congenital heart defects, the heart may be exposed to pathologically increased afterload [13]. Increased afterload reduces CFR in humans with aortic stenosis [14], and in dogs [15] and mice [10] with cardiac hypertrophy following aortic banding. Endothelium-dependent vasodilatation can be examined non-invasively in humans measuring flow mediated dilatation (FMD) of the brachial artery [16], [17], and several studies have evaluated FMD in pregnancy [18]–[24]. However, it is debated whether endothelial function in the peripheral vessels correlates with endothelial function in the coronary vascular bed [1], [17], [25], [26]. Furthermore, we are not aware of any published studies evaluating endothelial function in the coronary circulation during pregnancy, and to our knowledge the effect of increased afterload on CFR during pregnancy has not been reported.

The aims of this study were to (1) evaluate a non-invasive method of assessing CFR in rats using different concentrations of inhaled isoflurane for coronary vasodilation, (2) to investigate the differences in CFR between pregnant and non-pregnant rats and (3) to study how CFR is affected by increased afterload in pregnant and non-pregnant rats.

## Materials and Methods

### Ethics Statement

Animal experiments conformed to the Directive 2010/63/EU of the European Parliament on the protection of animals used for scientific purposes [27] and all procedures were approved by the Norwegian Committee on Ethics in Animal Experimentation (project ID 907 and 2177).

CFR was measured in young, female Wistar rats (Charles River, Sulzfeld, Germany) included in two experimental studies of increased cardiac afterload in pregnancy [28], [29]. In the first study (Ang II-study) mini osmotic pumps (Alzet Model 2002, Cupertino, CA, USA) releasing 150 ng/kg/min of angiotensin (Ang) II (Calbiochem, Darmstadt, Germany) or 0.9% sodium chloride was implanted subcutaneously in the neck of pregnant and non-pregnant rats for ∼10 days [28]. In the second study (TAC-study) transverse aorta constriction (TAC) or sham surgery was performed ∼14 days before the experiments in pregnant (on gestational day 5–8) and non-pregnant rats [29]. Both interventions led to increased afterload and cardiac hypertrophy with increased mean pressure in ascending aorta (AngII-study: 110±5 mmHg vs 84±5 mmHg, p = 0.001. TAC-study: 127±3 mmHg vs 96±5 mmHg, p<0.001) and heart weight (AngII-study: 896±30 mg vs 768±15 mg, p = 0.001. TAC-study: 845±29 mg vs 671±14 mg, p<0.001) as well as increased expression of B-type natriuretic peptide [28], [29]. In both studies the pregnant rats were examined close to term gestation (term = ∼21 days). Ten male rats were used to practice and evaluate the experimental procedure, dose of inhaled isoflurane and timing of CFR measurement using Doppler echocardiography. In four non-pregnant rats we compared the effect of 140 µg/kg/min continuous adenosine [8] infusion through the external jugular vein to 3.5% isoflurane inhalation.

**Figure 1:**
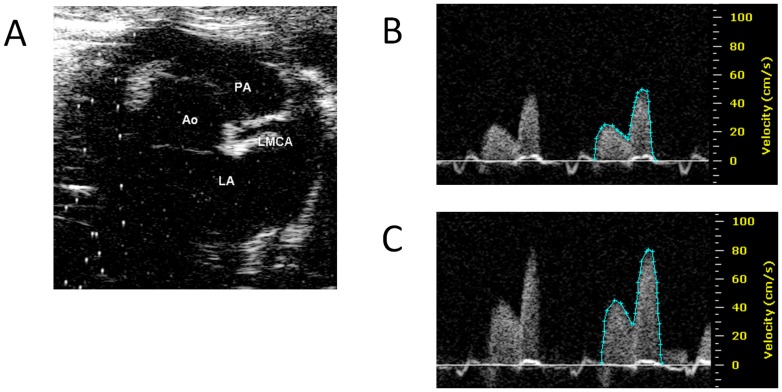
Measurement of coronary flow using Doppler echocardiography in rats. Parasternal short axis view of the heart (A) showing the Aorta (Ao), pulmonal artery (PA), left atrium (LA) and left main coronary artery (LMCA). Doppler velocity waveforms from the LMCA were traced and peak velocity and velocity time integrals were calculated during inhalation of 1.5% (B) and 3.5% (C) of isoflurane with oxygen.

Echocardiography was performed using a high resolution ultrasound imaging system equipped with a RMV-710B transducer with a frequency of 25 MHz and a fixed focal length of 15 mm mounted on an integrated rail system (Vevo 770, Visualsonics, Toronto, Canada) as described earlier [28], [29]. Anesthesia was induced with isoflurane 4% in 100% oxygen in an induction chamber. The rats were placed supine on a warm electric pad and inhaled isoflurane 1.5% in 100% oxygen was provided via a mask covering the rat's snout (Vevo Compact Anesthesia System, VisualSonics, Toronto, Canada) in spontaneously breathing rats (Ang II-study) or via an endotracheal tube in ventilated rats (TAC-study). The Doppler signals from the left main coronary artery (LMCA) was obtained from the parasternal short-axis view (Fig. 1A) by visualizing the aortic root and then moving the Doppler gate slightly anterior and to the left of the aorta. Doppler velocity waveforms with the highest peak flow velocity were recorded (Fig. 1B). Doppler signals could be detected even when the LMAC was not clearly visualized in B-mode echocardiography. After performing baseline measurements, the isoflurane concentration was increased to 3.5% and another recording of the LMCA Doppler signals was obtained after 2 minutes with the ultrasound probe fixed in the same position (Fig. 1C).

Echocardiography was performed without the knowledge of intervention, but the operator could not be blinded for pregnancy status. All ultrasound measurements were performed off-line without the knowledge of the animals' pregnancy status and intervention. Three consecutive cycles with good quality signals were measured and the average value was used for the statistics. Velocity-time integral (VTI) was outlined manually using the software provided with the ultrasound equipment (VisualSonics Vevo 770 v.3.0.0, Fig. 1BC), and peak velocity and VTI were calculated by the software. Angle correction was not used. CFR was calculated as the ratio between coronary flow velocities recorded at hyperemia (3.5% isoflurane) and at baseline (1.5% isoflurane). CFR_peak_ was calculated as peak velocity at hyperemia/peak velocity at baseline and CFR_VTI_ was calculated as VTI at hyperemia/VTI at baseline. Only echocardiographic examinations performed by a single operator were included in each study (Ang II-study: M.C.S., TAC-study: N.T.S.). After ultrasound, blood pressure (BP) was measured invasively via a 2F microtip pressure-volume catheter (SPR-838; Millar Instruments Inc, Houston, TX, USA) in the ascending aorta [28], [29].

### Statistics

Data are reported as mean±standard error of the mean (SEM). One way analysis of variance (ANOVA) was used to compare groups and the Holm–Sidak method was used as post-hoc test. Independent-Samples T-test was used to compare two groups. Correlation between parametric variables was checked using Pearson Correlation coefficient. A p-value of <0.05 was considered statistically significant. PASW Statistics 18.0.3 (SPSS Inc., Chigaco, IL, USA) software was used for statistical analyses.

## Results

**Figure 2:**
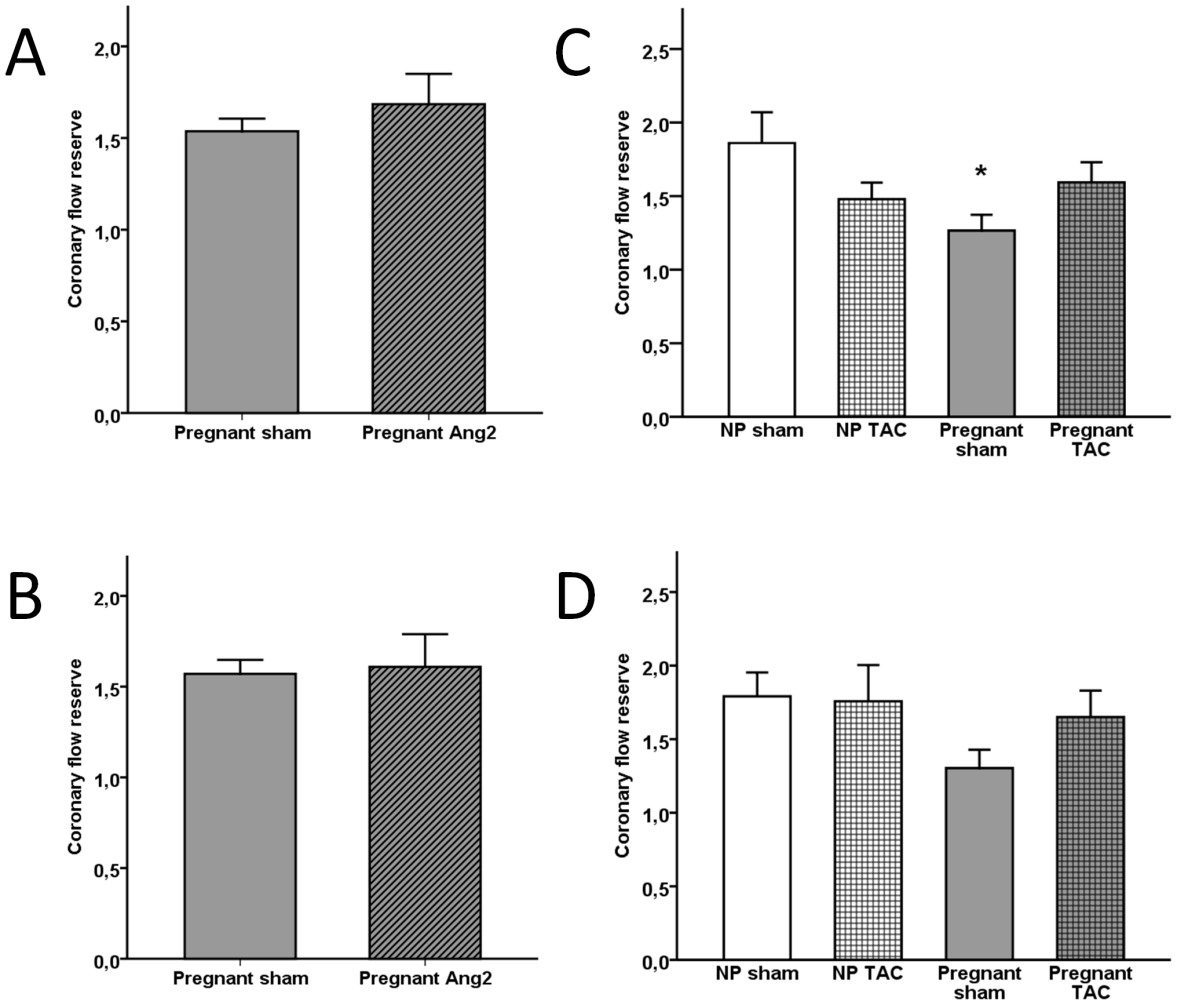
Coronary flow reserve in pregnant and non-pregnant rats. Mean ±SEM for coronary flow reserve calculated from peak velocities (A, C) and velocity time integrals (B, D). Comparisons between groups were made using independent samples T-test (A, B) and one-way ANOVA and Holm-Sidak post hoc test (C, D). SEM, standard error of the mean, NP, non-pregnant, Ang2, Angiotensin II, TAC, transverse aorta constriction, p<0.05 compared to non-pregnant sham (*).

In the Ang II-study we were able to obtain reliable Doppler flow velocity waveforms from the LMCA in 28 (85%) of 33 animals that had an echocardiography by the most experienced operator (M.C.S.); 13 pregnant sham, 11 pregnant Ang II, 2 non-pregnant sham, and 2 non-pregnant Ang II. Due to low numbers of non-pregnant animals, only data from pregnant animals are presented in Fig. 2. In the TAC-study echocardiography was performed by one operator (N.T.S.) in all animals. LMCA flow velocity was recorded in 36 of the 42 animals included. Four animals were excluded due to poor signal quality or inability to repeat measurements after increase of isoflurane concentration. Thus CFR measurements from 32 (76%) animals were eligible for analysis; 10 pregnant sham, 7 pregnant TAC, 7 non-pregnant sham, and 8 non-pregnant TAC.

Ten male rats were used to evaluate the effect of inhaled isoflurane on coronary flow. Coronary flow velocities increased and stabilized one to two minutes after the concentration of inhaled isoflurane was increased from 1.5% to 3.5%, and returned to baseline values three to five minutes after the isoflurane concentration was reduced to 1.5%. Increase in inhaled isoflurane concentration above 3.5% in four rats did not lead to further increase in coronary flow velocities, and there was no difference in calculated CFR_peak_ or CFR_VTI_ at 3.5% compared to 4.5% (two rats) or 5.0%(two rats) concentration of inhaled isoflurane (CFR_peak_:1.78±0.13 vs 1.63±0.11, p = 0.4. CFR_VTI_: 1.96±0.29 vs 2.05±0.27, p = 0.3). In most animals measurement of coronary flow was not feasible when the inhaled isoflurane concentration was below 1.5% as the animals would start moving and the coronary flow signals were lost. In two rats blood flow velocities were similar at 1.0% and 1.5% isoflurane.

Adenosine infusion led to a significant increase in coronary flow velocity only in one of four rats. In this rat the calculated CFR_peak_ was similar following adenosine infusion and isoflurane inhalation (1.82 and 1.89 respectively). In three rats not responding to adenosine the calculated CFR_peak_ following isoflurane inhalation was 1.11, 1.67 and 1.72.

**Figure 3:**
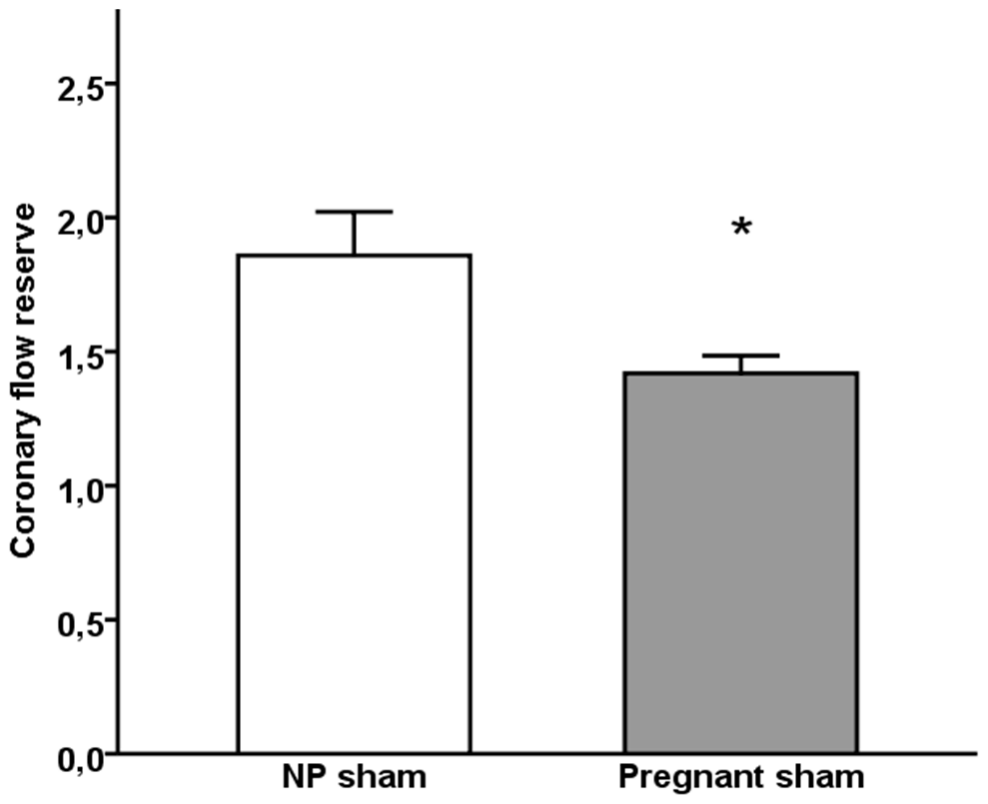
Coronary flow reserve in sham rats. Mean ±SEM for coronary flow reserve calculated from peak velocities in non-pregnant (NP) and pregnant sham animals from both studies. Independent-Samples T-test was used to compare groups. SEM, standard error of the mean, NP, non-pregnant, p<0.05 compared to non-pregnant sham (*).

Calculated CFR_peak_ and CFR_VTI_ values are presented in Fig. 2. There were no differences in CFR_peak_ or CFR_VTI_ between pregnant sham and pregnant AngII-infused rats. In the TAC-study CFR_peak_ (1.54±0.07 vs 1.85±0.17, p = 0.03) but not CFR_VTI_ (1.57±0.08 vs 1.72±0.06, p = 0.3) was decreased in pregnant compared to non-pregnant sham. TAC did not significantly affect CFR_peak_ or CFR_VTI_ in pregnant or non-pregnant animals. When pregnant (n = 23) and non-pregnant (n = 9) sham animals from both studies were pooled together and compared, both CFR_peak_ (1.42±0.07 vs 1.86±0.16, p = 0.005, Fig. 3) and CFR_VTI_ (1.45±0.07 vs 1.78±0.12; p = 0.03) were significantly decreased in pregnant animals. There was no correlation between CFR_peak_ and systolic or diastolic BP, heart weight or left ventricular contractility measured as left ventricular fractional shortening by m-mode echocardiography.

## Discussion

We measured CFR non-invasively in pregnant and non-pregnant rats using high concentrations of inhaled isoflurane as a vasodilating agent. We were able to calculate CFR using Doppler flow velocity waveforms of LMCA in most animals. CFR was reduced in pregnant rats compared to non-pregnant. Furthermore, we investigated whether increased afterload has an effect on CFR in pregnant and non-pregnant rats and found that neither chronic Ang II infusion nor aorta constriction affected CFR.

In the TAC-study CFR_peak_ was reduced in pregnant compared to non-pregnant sham operated rats. The same trend was observed in sham treated rats in the AngII-study (Fig. 2A), however only two non-pregnant rats were examined and difference was not statistically significant. Combining data on sham animals from two studies, we found that both CFR_peak_ and CFR_VTI_ were significantly lower in pregnant compared to non-pregnant rats. There are no published studies on CFR in pregnancy. FMD reflects the endothelial function in the peripheral resistance vessels but may not correlate with that of the coronary vascular bed [17], [25], [26], [30]. Studies in pregnant women have shown that FMD decreases towards term [19], [20], [23] which is in line with our findings.

To our knowledge, the effects of pregnancy on CFR have not been studied before, and it is not known if reduced CFR increases the risk of adverse cardiovascular events in pregnancy. A recent study showed that the utero-placental blood flow is impaired in pregnant women with congenital heart diseases [31].The course and outcome of pregnancy may be affected by maternal heart disease [32]. Death from heart disease is the leading cause of indirect maternal death in the UK [33]. Hirata et al. have shown an increase in CFR associated with increasing levels of 17β-estrogen in the follicular phase of the menstrual cycle in young, healthy women and after administration of conjugated estrogen in postmenopausal women [34]. Profound hormonal changes that occur during pregnancy could be responsible for reduced CFR that we observed in rats close to term. There is a need for non-invasive tests to asses risk and predict outcome in pregnant women at risk. Thus, studies of CFR in healthy human pregnancies and in pregnant women with hypertension or heart diseases using non-invasive methods and safe vasodilating agents (e.g. dipyridamole, a FDA category B drug) are warranted.

In contrast to previous findings in dogs [15] and mice [10] with increased afterload, in our study CFR was not reduced by Ang II infusion or TAC in rats. In addition to species differences, there could be several other reasons for this discrepancy. Hittinger et al. found that CFR was preserved in dogs with compensated heart hypertrophy, but was exhausted when the dogs developed decompensated pressure overload left ventricular hypertrophy [15]. In our studies the animals did not have decompensated heart failure when CFR was measured [28], [29]. The coronary circulation is subjected to autoregulation and coronary blood flow is constant over a wide range of pressures, referred to as the ‘zero pressure flow phenomenon’ [1], [35]. When the vascular bed of the heart is maximally vasodilated (e.g. by adenosine or isoflurane) the coronary autoregulation is revoked and coronary blood flow exhibits a linear relationship with the myocardial perfusion pressure [1], [36]. Therefore, a change in blood pressure will affect CFR. As coronary flow is predominantly diastolic in the rat LMCA (Fig. 1BC), an increase in diastolic BP may increase calculated CFR. In our study, systolic rather than diastolic BP was increased following TAC and the pregnant sham animals had a significantly lower diastolic BP than all other groups [29]. Therefore, the reduced CFR in late pregnancy may be a result of reduced diastolic BP rather than impaired coronary endothelial function. Examining CFR and BP at mid-term could have added valuable information, but we refrained from anaesthetising the animals twice during pregnancy to avoid adverse effects on mother and fetuses.

In contrary to TAC, chronic Ang II infusion led to significantly increased diastolic as well as systolic pressure [28]. Furthermore, Ang II infusion led to significant fibrosis of the heart tissue, whereas TAC did not [28], [29]. Thus the Ang II infused rats probably were closer to heart failure. According to the ‘zero pressure flow phenomenon’ an increased diastolic BP in Ang II infused rats will increase calculated CFR and may mask an actual impairment of the coronary vessels ability to dilate [1], [35]. Hoffmann et al. defined CFR as the maximal increase in coronary blood flow above its basal level for a given perfusion pressure when coronary circulation is maximally dilated [37], and ideally speaking, perfusion pressure needs to be taken into account while comparing coronary flows in different animals. Thus, even though the CFR was not significantly affected by Ang II infusion, we cannot conclude that coronary microvascular dilatation is not impaired by Ang II.

The gold standard method for measuring CFR is by using adenosine infusion. We used inhaled isoflurane which is commonly used as a safe anesthetic in rats. This method is non-invasive and does not require insertion of an intravenous cannula and is more practical. We found that this method of measuring CFR in rats using different concentrations of inhaled isoflurane and echocardiography is feasible in a laboratory setting. The operator of the Ang II-study was more experienced in small-animal echocardiography than the operator of the TAC-study and had a higher success rate in obtaining reliable CFR measurements. However, the difference between operators was not significant (p = 0.4). The most frequent cause of not obtaining CFR measurements was inability to detect LMCA flow velocity signals consistently. Using an ultrasound system equipped with color Doppler could facilitate the identification of coronary artery, and increase the success rate.

3.5% of isoflurane was chosen as high-concentration as this was set as the maximum maintenance dose used in rats in our laboratory. High concentrations of inhaled isoflurane may lead to reduced cardiac output, reduced BP, circulatory collapse and cardiac arrest. In four male rats an increase in isoflurane concentration above 3.5% did not lead to further increase in coronary flow velocity indicating that the coronary vessels were maximally dilated at 3.5%.

Due to the small caliber of coronary artery and limited image resolution, its diameter is difficult to measure and coronary volume blood flow cannot be calculated non-invasively. However, coronary flow reserve estimated using Doppler flow velocity waveforms correlates well with invasively measured CFR [38]. Previous studies mostly report CFR_peak_ rather than CFR_VTI_. We have presented both the CFR_peak_ and CFR_VTI_ in our results.

An increase in peak velocity during the diastole is expected following coronary vasodilatation. However, an increase in systolic component was also observed during hyperemia (Fig. 1BC). Therefore measuring VTI of the flow velocity waveform during the whole cardiac cycle might be a better estimate of coronary flow than just the peak velocity.

## Conclusions

It is feasible to measure CFR in rats non-invasively using Doppler echocardiography and high concentration of inhaled isoflurane as the vasodilating agent. CFR was not affected by increased left ventricular cardiac afterload caused by chronic Ang II infusion or TAC both in pregnant and non-pregnant rats. CFR is reduced in late pregnancy (close to term) in rats. Whether this reduction is a result of impaired coronary endothelial function or is merely a reflection of reduced diastolic BP in late pregnancy remains to be elucidated.
