# Correction: Coronary Flow Reserve in Pregnant Rats with Increased Left Ventricular Afterload

**DOI:** 10.1371/journal.pone.0111272

**Published:** 2014-10-17

**Authors:** The PLOS ONE Staff

The fourth author’s name is spelled incorrectly. The correct name is David Johansen. The correct citation is: Songstad NT, Serrano MC, Sitras V, Johansen D, Ytrehus K, et al. (2014) Coronary Flow Reserve in Pregnant Rats with Increased Left Ventricular Afterload. PLoS ONE 9(7): e102147. doi:10.1371/journal.pone.0102147.
